# Electrode Materials for Glyphosate Removal from Water by Advanced Anodic Oxidation Processes: A Critical Review

**DOI:** 10.3390/ma19122578

**Published:** 2026-06-15

**Authors:** Wiyao Maturin Awesso, Sophie Tingry, Akpénè Amenuvevega Dougna, Ibrahim Tchakala, Seyf-Laye Alfa-Sika Mande, Marc Cretin

**Affiliations:** 1 Institut Européen des Membranes (IEM), UMR 5635, University of Montpellier, ENSCM, CNRS, 34090 Montpellier, France; awessomaturin0@gmail.com (W.M.A.); sophie.tingry@umontpellier.fr (S.T.); 2 Laboratory of Water Resources and Environmental Engineering, Faculty of Sciences and Technology, University of Kara, Kara BP 404, Togo; adougna@yahoo.fr (A.A.D.); itchakala@univ-lome.tg (I.T.); seyf009@yahoo.ca (S.-L.A.-S.M.); 3 Applied Hydrology and Environmental Laboratory (Formerly Water Chemistry Laboratory), University of Lomé, Lomé BP 1515, Togo; 4 Beijing Key Laboratory of Water Resources Environmental Engineering, China University of Geosciences (Beijing), Beijing 100083, China

**Keywords:** glyphosate, electrochemical anodic oxidation, hydroxyl radicals, electrode materials, water contamination, mineralization

## Abstract

**Highlights:**

- Electrochemical anodic oxidation is critically reviewed as an efficient advanced oxidation process for glyphosate removal from contaminated waters.
- The role of anode materials (BDD, PbO_2_, mixed metal oxides and Magnéli-phase Ti_4_O_7_) in controlling degradation, mineralization and energy efficiency is systematically compared.
- Hydroxyl radicals generated at high oxygen evolution overpotential anodes are identified as the main reactive species driving glyphosate mineralization.
- Operating parameters such as current density, pH and electrolyte composition strongly influence kinetics, by-product formation and current efficiency.
- Magnéli-phase Ti_4_O_7_ electrodes emerge as a cost-effective and promising alternative to BDD, especially when integrated with membrane processes for treating concentrated effluents.

**Abstract:**

Glyphosate, the most extensively used herbicide worldwide, is frequently detected in aquatic environments due to its high solubility, persistence, and intensive agricultural application. Its occurrence, together with that of its principal metabolite aminomethylphosphonic acid (AMPA), raises substantial environmental and public health concerns. Conventional water treatment technologies generally exhibit limited efficiency in achieving complete removal and mineralization of this compound. In recent years, advanced electrochemical oxidation processes, and particularly anodic oxidation, have emerged as promising alternatives owing to their ability to generate highly reactive hydroxyl radicals in situ. This review provides the first contaminant-specific and mechanistic assessment dedicated exclusively to the anodic electro-oxidation of glyphosate. In contrast to previous reviews offering broad surveys of electrode materials or generalized evaluations of glyphosate treatment technologies, this work synthesizes all mechanistic, kinetic, and material-dependent insights reported between 2016 and 2025. A comparative analysis of major anode families (including boron-doped diamond (BDD), ${PbO}_{2}$, mixed-metal oxides, and Magnéli-phase Ti_4_O_7_) is presented, highlighting glyphosate-specific degradation pathways, intermediate formation, and the operational parameters controlling mineralization efficiency and energy demand. By establishing a structured framework that links electrode properties, radical-generation mechanisms, and pollutant-specific degradation chemistry, this review addresses a critical gap in the literature and provides a scientific basis for designing next-generation electrochemical processes for the efficient and sustainable removal of glyphosate and related organophosphorus contaminants.

## 1. Introduction

The growing global demand for food, driven by population growth, has led to an intensification of agricultural practices and increased the use of agrochemicals, resulting in significant environmental and societal impacts. One major concern is water pollution caused by pesticides, given their potentially harmful effects on ecosystems and human health [1]. Among these chemicals, herbicides are the most widely used in agricultural areas to eliminate or inhibit weed growth in crops. Since 1974, glyphosate-based N-(phosphonomethyl)glycine herbicides, classified as highly effective, non-selective anionic herbicides, have become the most extensively applied. In EU countries, it accounts for more than 72% of total pesticide use [2,3,4,5] and 33% of all herbicide sales, making it one of the most widely used herbicides in Europe [6]. Glyphosate ($C_{3}H_{8}{NO}_{5}P$) is a water-soluble herbicide, present in groundwater at concentrations of up to 24 ${\mu g\;L}^{-1}$ and in surface water at levels reaching 50 ${\mu g\;L}^{-1}$ [7,8]. Its half-life in soil ranges from 2 to 215 days, depending on soil properties (such as pH, texture, mineral composition and organic matter), while in aquatic environments it ranges from 2 to 91 days. The primary degradation product of glyphosate, aminomethylphosphonic acid (AMPA), is also found in plants, water, and soil. AMPA exhibits lower water solubility (5.8 ${µg\;L}^{-1}$ at 25 °C) and a longer half-life in soil, ranging from 60 to 240 days [5,6].

Due to its persistent presence across various environments such as water, soil and air, this herbicide and its metabolites are recognized as probably being implicated in the development of cardiovascular, digestive, pulmonary and immunological disorders [5]. Regarding human health risks, the World Health Organization and the International Agency for Research on Cancer have classified the herbicide glyphosate and its primary metabolite, AMPA, as probably carcinogenic to humans and of potential toxicological concern. This classification is mainly attributed to their residual accumulation in the food chain and the possible contamination of drinking water sources [9]. In the USA, where glyphosate consumption accounts for 19% of global consumption, glyphosate and AMPA has been detected in most stream and river samples, with concentrations reaching up to 430 ${µg\;L}^{-1}$ [10], requiring the development of methods for its removal from water.

Given the health and environmental risks associated with aquatic environments [11,12,13], both biological and physico-chemical processes have been developed to treat water polluted by glyphosate, its co-adjuvants and metabolites [14,15] (Table 1). These treatment methods each present specific advantages and limitations in terms of efficiency, cost, feasibility, and environmental impact. Physicochemical processes (such as coagulation, adsorption, and reverse osmosis) [16] are non-destructive and therefore do not result in total destruction of the organic contaminants, often requiring expensive post-treatment steps [17,18]. Conversely, biological processes based on enzymatic reactions cannot achieve high mineralization efficiency, because microorganisms convert glyphosate primarily into intermediate metabolites (AMPA and/or sarcosine) rather than fully decomposing it to CO_2_ and inorganic ions, and are heavily dependent on specific microorganism growth conditions [19,20].

In addition to these conventional methods, advanced oxidation processes have emerged over the past 25 years as an effective approach for water treatment (Table 1). These processes rely on the generation of highly reactive oxidizing species in solution -such as hydroxyl radicals (^•^OH)-through electrochemical or photochemical reactions, capable of degrading organic matter to complete mineralization [21,22,23,24]. These processes generally operate at near-ambient temperature and pressure and have received considerable attention at both the laboratory and pilot scales. Their main advantage lies in their ability to eliminate persistent compounds at concentrations ranging from several hundred ppm to below 5 ppb, achieving removal efficiencies exceeding 90%. In his review of 2014 [25], P. Chaplin describes the various mechanisms involved in the oxidation of organic compounds during these processes, and provides a critical analysis of published research. The review also highlights the challenges that must be addressed for these technologies to be fully implemented in water treatment. More recently, in 2024, V.B.K. Yaah et al. [15] have reviewed the various technologies developed since 2012 for glyphosate removal from water, including the advanced oxidation processes such as photocatalysis, Fenton reaction, and ozonation. Their work discusses the associated challenges and provides real-world application examples.

Among the advanced oxidation processes, electrochemical anodic oxidation process relies primarily on the in situ generation of reactive radicals, such as ^●^OH, ${SO}_{4}^{•-}$, ${CO}_{3}^{•-}$, and ${Cl}^{•}$, capable of partially or completely oxidizing organic or organometallic pollutants to their final mineralization forms (${CO}_{2}$ and $H_{2}O$ at least) [26]. This process works under ambient conditions without the addition of other chemical agents to carry out the degradation and offers the advantage of easy automation and control. However, as with other electrochemical processes, its efficiency, durability and cost largely depend on the nature of the anode material where the radicals are produced, which is subject to polarization, passivation and corrosion. Among the radicals generated at the anode surface, ^●^OH is considered the most powerful radical (redox potential E° = 2.80 V/ESH) produced directly from water oxidation without the addition of chemicals [27]. Relevant works have focused on identifying and quantifying ^●^OH in anodic oxidation systems by exploring different methods, especially those used to compare the electrochemical performance of commonly used anodes [28,29]. The current detection methods for free radicals are predominantly indirect and fall into two main categories. The first approach uses chemical reaction-based techniques that degrade and transform specific compounds via free radicals, often paired with advanced analytical tools (e.g., HPLC, spectrophotometry, chemiluminescence, gas chromatography, fluorescence spectrometry) to identify and indirectly quantify free radical concentrations. The second category, known as the capture method, employs spin trapping agents to stabilize free radicals by forming spin adducts. These adducts are then detected using sophisticated technologies like electron spin resonance (ESR), also known as electron paramagnetic resonance (EPR) spectroscopy.

The formation rate and concentration of free radicals in anodic oxidation processes are influenced by a variety of operational parameters that play a significant role, including the applied current, the pH of the solution, and the electrolyte composition [30]. However, as the radicals are typically produced at the surface of the anode through electrochemical reactions, consequently, the choice of anode material is a key determinant of the overall oxidation efficiency of the process. The interaction between anode material and operating conditions strongly influences the stability and reactivity of generated radicals. Understanding and optimizing these parameters is thus crucial for enhancing the performance of anodic oxidation processes in the removal of persistent organic pollutants. The desired properties of anode materials include a high potential for oxygen evolution reaction (OER), good electrochemical stability, long service life, cost-effectiveness, and environmental friendliness. In their review [30] M. Shestakova et al. provide a broad and valuable overview of electrode materials used in the electrochemical oxidation of organic compounds. The contribution focuses primarily on categorizing electrode families (BDD, ${PbO}_{2}$, MMO, carbon-based electrodes, etc.) and summarizing their general properties, stability issues, fabrication methods, and performance trends across a wide variety of organic pollutants. The discussion is intentionally generic and does not address the specific mechanistic, kinetic, or operational challenges associated with individual contaminants, particularly glyphosate, which is mentioned only marginally. Other reviews have examined glyphosate degradation via hydroxyl radicals generated through alternative chemical or electrochemical methods, such as photoelectrochemical and electro-Fenton processes, without addressing electrochemical anodic oxidation [31,32,33].

The present work distinguishes itself through its substantial originality, as it constitutes the first comprehensive, critically organized review dedicated exclusively to the anodic electro-oxidation of glyphosate. Unlike previous review articles, which provided broad overviews of wastewater-treatment technologies without undertaking a detailed analysis of electrochemical degradation pathways [16,33], this review offers a mechanistic and materials-oriented examination of the process. It systematically assesses the physicochemical properties and electrocatalytic behavior of widely used anodic materials, elucidates the fundamental oxidation routes and key intermediates, and evaluates the influence of operational parameters (including current regime, applied potential, mass-transfer conditions, and electrolyte composition) on degradation kinetics, mineralization efficiency, and energy requirements.

In addition, the review integrates and synthesizes advances published between 2016 and 2025, which were not covered in earlier state-of-the-art assessments. The comparative framework developed herein enables rigorous benchmarking of electrode performance in the precise context of glyphosate degradation, the identification of intrinsic and extrinsic rate-limiting factors, and the delineation of dominant anodic oxidation mechanisms across material families (boron-doped diamond, mixed-metal oxides, doped carbon materials, and emerging hybrid architectures), absent from generic electrode reviews. This analytical approach highlights the main technological bottlenecks currently limiting the electro-oxidation of glyphosate (such as high energy consumption, formation of persistent transformation products, and long-term electrode stability) and proposes rational strategies to address them.

Overall, this review fills a critical gap in the literature by providing the first dedicated, mechanism-focused, and materials-centered evaluation of glyphosate electro-oxidation. It establishes a coherent methodological framework to support the development of next-generation electrochemical treatments with improved efficiency, selectivity, and sustainability for the degradation of glyphosate and related organophosphorus contaminants, while offering practical guidance for future research directions and for the optimization of this advanced treatment technology.

## 2. Anodic Electro-Oxidation Process Applied to the Removal of Glyphosate

Advanced oxidation processes (AOPs) applied to glyphosate removal are characterized by the in situ generation of highly reactive oxidizing species, particularly hydroxyl radicals (^●^OH), which can rapidly and efficiently degrade most organic compounds up to complete mineralization into CO_2_, H_2_O, and inorganic ions [34,35]. With a redox potential of 2.8 V vs. SHE in acidic solution, ^●^OH is significantly more reactive than conventional oxidants such as chlorine or ozone.

**Figure 1:**
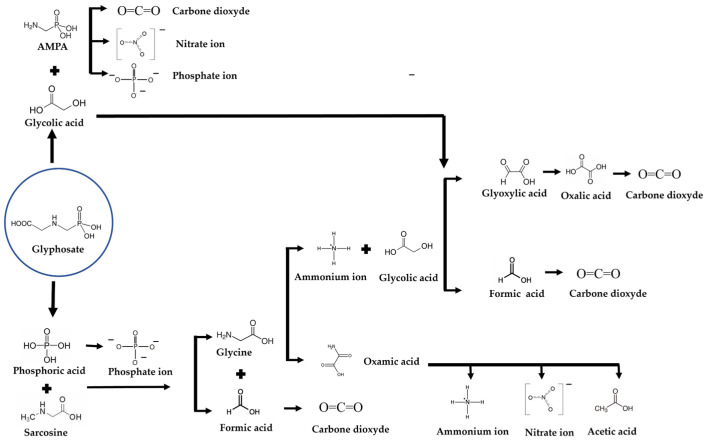
Proposed degradation pathway of glyphosate by electro-oxidation. (Adapted from an open-source journal of MDPI [38]).

In the case of glyphosate, oxidative degradation driven by hydroxyl radicals ^●^OH leads primarily to the cleavage of C–N and C–P bonds, resulting in the formation of intermediates such as AMPA, sarcosine, or glycine, or, in the case of complete mineralization, final products including nitrate, ammonium, phosphate ions, along with carbon dioxide, and water [33] (Equation (1)). The identification of these intermediate and final products, typically achieved through chromatographic analyses, enables the elucidation of degradation pathways that may occur independently or simultaneously during the electrooxidation process (Figure 1) [36,37,38].

(1) $${2C}_{3}H_{8}{NO}_{5}P+38^{●}\mathrm{OH}\;\to \;6{CO}_{2}+25H_{2}O+{NO}_{3}^{-}+{NH}_{4}^{+}+{2PO}_{4}^{3-}$$

^●^OH can be formed by catalytic, electrochemical and/or photochemical activation. Among the strategies for glyphosate removal from water, there are photooxidative methods, including photocatalytic degradation [39,40,41], UV-assisted oxidation [42,43], Fenton/electro-Fenton/Photo-Fenton [44,45,46], ozonation [21,34] and electrochemical oxidative approaches [47]. The main advanced oxidation processes for glyphosate removal, along with their respective advantages and limitations, are summarized in Table 2. Electrochemical oxidation is particularly suitable for glyphosate treatment, a highly recalcitrant organophosphorus compound, due to its chemical-free operation and high mineralization capability, although its performance may be limited by mass transfer constraints and electrode stability issues [47]. Ozonation and photo-assisted processes are effective but restricted by the low solubility of ozone and scale-up limitations, respectively. Fenton-based processes show high efficiency at low pollutant concentrations but require acidic pH conditions and generate secondary sludge [48]. In this context, anodic electro-oxidation emerges as a robust and efficient technology for glyphosate degradation. In addition, hybrid processes combining two or more treatment methods to enhance degradation efficiency are also considered in this comparison. ^●^OH can be formed by catalytic, electrochemical and/or photochemical activation. Among the strategies for glyphosate removal from water, there are photooxidative methods, including photocatalytic degradation [39,40,41], UV-assisted oxidation [42,43], Fenton/electro-Fenton/Photo-Fenton [44,45,46], ozonation [21,34] and electrochemical oxidative approaches [47]. The main advanced oxidation processes for glyphosate removal, along with their respective advantages and limitations, are summarized in Table 2. Electrochemical oxidation is particularly suitable for glyphosate treatment, a highly recalcitrant organophosphorus compound, due to its chemical-free operation and high mineralization capability, although its performance may be limited by mass transfer constraints and electrode stability issues [47]. Ozonation and photo-assisted processes are effective but restricted by the low solubility of ozone and scale-up limitations, respectively. Fenton-based processes show high efficiency at low pollutant concentrations but require acidic pH conditions and generate secondary sludge [48]. In this context, anodic electro-oxidation emerges as a robust and efficient technology for glyphosate degradation. In addition, hybrid processes combining two or more treatment methods to enhance degradation efficiency are also considered in this comparison.

The literature provides relevant comparisons of these methods in terms of glyphosate removal performance depending on operating conditions (such as pH, catalyst quantity, current density, UV lamp wavelength, glyphosate concentration) [21,46,49,50,51].

Among advanced oxidation processes, electrochemical anodic oxidation operates under controlled energy input at the anode to decompose organic compounds into stable mineral molecules and/or simpler structures that are more easily biodegradable. When applied to water purification, this method operates through two fundamental mechanisms that govern pollutant degradation: (1) direct oxidation, where pollutants migrate toward and adsorb onto the anode surface and undergo electron transfer reactions. However, this process often leads to partial degradation rather than complete mineralization, as the reaction is limited to species in direct contact with the electrode; (2) indirect oxidation, a more powerful pathway that enables extensive pollutant removal. In this mechanism, reactive species such as ^●^OH and other highly oxidative oxygen species are generated electrochemically during water dissociation at the anode. These species diffuse into the bulk solution and attack pollutants, promoting deep oxidation reactions that can ultimately convert organic contaminants into harmless end products like CO_2_ and water. Indirect oxidation is particularly advantageous because it does not rely on direct contact between pollutants and the electrode surface. Instead, it leverages the high reactivity of transient radicals and oxygen species, enabling uniform treatment throughout the solution and achieving complete mineralization under optimized conditions [35]. Indirect oxidation involves oxidative species electrochemically generated during water dissociation at the anode (Equation (2)).

Direct oxidation is favored at anode materials (M) characterized by a low oxygen overpotential, such as Pt, ${RuO}_{2}$, or ${IrO}_{2}$, which interact strongly with ^●^OH radicals, resulting in transformation into higher oxide or superoxide chemisorbed on the anode surface (MO) (Equation (3)). It allows only partial oxidation of organics (R), forming some organic compounds (RO) such as short carboxylic acids and other degradation intermediaries, and depends on the complexity and stability of pollutants and treatment conditions (Equation (4)) [52].

(2) $$M+H_{2}O\;\to \;{(M)\;}^{●}\mathrm{OH}+H^{+}+e^{-}$$

(3) $$(M{)}^{•}\mathrm{OH}\;\to \;\mathrm{MO}+H^{+}+e^{-}$$

(4) MO+R → M+RO

In contrast, indirect oxidation occurs at anode materials, such as ${PbO}_{2}$, ${SnO}_{2}$, ${Ti}_{4}O_{7}$ and boron-doped diamond (BDD), where physiosorbed hydroxyl radicals (M(^●^OH)) are produced via water oxidation (Equation (2)). These radicals are weakly adsorbed and highly reactive, enabling the complete mineralization of organic pollutants. Due to the inert nature of these electrode surfaces, they do not facilitate the adsorption of organic compounds and function solely as electron acceptors [52].

**Figure 2:**
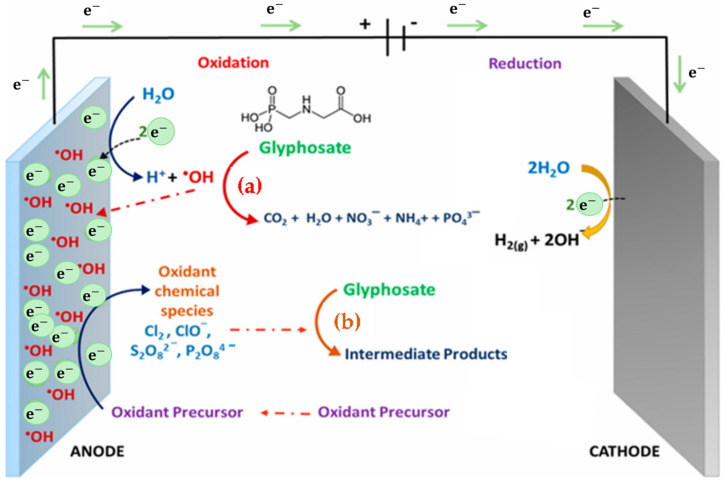
Oxidation mechanisms of glyphosate molecules at anodes with high oxygen overpotential via (**a**) ^●^OH and (**b**) oxidant chemical species from inorganic ions present in the solution. (Adapted from an open-source journal of MDPI [33]).

Among ^●^OH formation at the electrode surface, indirect oxidation of micropollutants has also been observed via other reactive oxygen species (such as ${H_{2}O}_{2}$ or $O_{3}$), which are produced in situ at the electrode surface at potentials above 2.3 V/SHE, as well as through reactive species formed from electrolyte-derived inorganic ions, including chloride, persulfate ($S_{2}O_{8}^{2-}$), carbonate (e.g., ${CO}_{3}^{•-}$), and pyrophosphate ($P_{2}O_{8}^{4-}$) [53]. While chlorine-mediated oxidation is largely independent of electrode material, the generation of other reactive oxygen species (ROS) is more efficient at anodes with high oxygen evolution overpotentials. Figure 2 illustrates the anodic degradation pathways of glyphosate in aqueous media: (a) via ^●^OH generated from water oxidation, and (b) via oxidants derived from electrolyte ions.

## 3. Factors Influencing the Efficiency of the Anodic Electrooxidation Process

In electrochemical processes, the efficiency of pollutant degradation is primarily influenced by the nature of the electrode, alongside several other operational factors, including current density, the type and concentration of the supporting electrolyte, mass-transfer limitations, solution pH, and the purity of the reagents used. Each of these parameters plays a critical role in determining the rate and extent of electrochemical reactions, thereby affecting the overall performance of the treatment process. This section provides a brief overview of the general trends associated with these operational variables.

According to the literature, hydroxyl radicals are the primary agents responsible for glyphosate degradation during electrochemical oxidation, ensuring consistent and effective mineralization. Two key characteristics of this process emerge: (1) the degradation rate is limited by mass transport, and (2) the reaction follows pseudo-first-order kinetics. For pesticide degradation, mass transport is controlled by the diffusion of contaminants toward reactive sites near the anode. This limitation is evident from the nonlinear relationship between applied current and degradation rate, with several studies reporting a plateau at higher currents. Current efficiency (defined as the ratio of observed degradation to the theoretical value based on charge consumption) typically decreases as current increases, indicating the occurrence of side reactions. The pseudo-first-order behavior results from the interplay between oxidant generation, contaminant degradation, and diffusion through the layer near the electrode surface. Despite glyphosate being present at much higher concentrations than hydroxyl radicals generated at a given current, the overall process remains diffusion-limited, with the rate primarily governed by the transport of contaminants to the electrode surface.

### 3.1. Operating Parameters

➢ Effect of current density

For all reviewed literature, increasing current density enhanced both initial degradation and mineralization rates. This effect is primarily attributed to elevated ^●^OH generation and the formation of secondary oxidants from electrolyte species (e.g., sulfate, phosphate, chloride) [54]. However, the degradation rate increase is not proportional to the current, due to mass transport limitations. As the current rises, competing parasitic reactions such as $H_{2}O_{2}$, chlorate, $O_{2}$, and $O_{3}$ formation become more prominent, reducing current efficiency and limiting further improvements in degradation performance [54]. In addition, application of high current densities implies higher energy requirements that unnecessarily increase operational costs.

➢ Effect of pH

pH is a critical parameter in wastewater treatment to define the operational boundaries for emerging technologies. Glyphosate, an aminophosphonic acid, exhibits multiple ionic species due to its acid dissociation constants (pKa1 = 0.78 for the phosphonate group and pKa2 = 2.29 for the carboxyl group) [55]. As a result, these zwitterionic forms display distinct electrochemical reactivity, which significantly affects the kinetics and overall efficiency of electrochemical oxidation [56]. However, the predominant influence of pH lies in its effect on hydroxyl radical (^●^OH) generation. Acidic conditions enhance glyphosate degradation by promoting ^●^OH formation and facilitating physisorption onto the anode surface. In contrast, higher pH levels reduce current efficiency for ^●^OH production, favoring the oxygen evolution reaction (OER) and thereby diminishing degradation rates. Additionally, pH can modulate the formation of reactive oxidant species derived from the supporting electrolyte, further impacting the degradation pathway and overall treatment performance.

➢ Effect of oxidative species evolution

Electrolyte composition can significantly influence the mineralization rate of glyphosate during electrochemical oxidation, primarily due to the electrogeneration of distinct oxidant species, as evidenced by variations in total organic carbon (TOC) removal. Most studies employed low-concentration electrolytes (~50 mM), typically NaCl, ${Na}_{2}{SO}_{4}$, ${Na}_{2}{CO}_{3}$ or $H_{2}{SO}_{4}$. Generally, higher electrolyte concentrations improved glyphosate removal, with increased oxidant generation. These oxidants, more stable than hydroxyl radicals, can diffuse beyond the electrode surface, contributing to bulk-phase oxidation.

The effect of electrolyte nature on glyphosate degradation is related in several studies. The reaction rate constants are generally highest with ${Na}_{2}{SO}_{4}$ as the supporting electrolyte, attributed to the formation of persulfate ($S_{2}O_{8}^{2-}$) oxidants [57]. While ${Na}_{2}{SO}_{4}\;$ favored complete mineralization, NaCl shows faster initial degradation rates. However, increased NaCl concentrations led to the formation of recalcitrant chlorinated by-products, reducing overall mineralization. Chloride ions also produce a broader range of intermediates and potential disinfection by-products, highlighting the need to balance degradation efficiency with by-product formation [58].

➢ Technical performance parameters

Technical performance parameters are essential analytical tools for assessing the performance of electrochemical technologies, particularly in terms of efficiency and energy consumption, both of which directly impact operational costs. In the context of electrochemical oxidation, mineralization current efficiency (MCE) serves as a key indicator for evaluating TOC removal. MCE quantitatively reflects the proportion of electrons effectively utilized in mineralization reactions that contribute to the reduction in total organic carbon, thereby offering insight into the process’s electrochemical efficiency [59,60,61]. Another parameter is energy consumption, which quantifies the energy required to achieve mineralization of the total organic carbon (TOC) load, as defined by the corresponding expression. Assessing these parameters enables the identification of optimal operating conditions for glyphosate degradation that maximize mineralization current efficiency (MCE) while minimizing energy consumption.

### 3.2. Electrode Material Under Specific Operating Conditions

The nature of the electrode material strongly influences the selectivity and efficiency of the electro-oxidation process, favoring either the partial and selective oxidation of pollutants or complete combustion into ${CO}_{2}$ [33]. The ideal anode material should promote the generation of weakly adsorbed ^●^OH while exhibiting a high potential for the oxygen evolution reaction. In practice, however, most anodes exhibit intermediate behavior, with organic oxidation and oxygen evolution reactions occurring simultaneously via parallel reaction pathways.

This section examines the degradation and mineralization performance of glyphosate and the associated mechanisms, with a particular focus on the nature of the most effective anodes employed in electrochemical advanced oxidation processes. It should be noted that quantitative long-term stability data and standardized techno-economic evaluations of electrode materials remain scarce and highly heterogeneous in the literature [62,63,64]; therefore, only qualitative comparisons based on reported trends are considered in this review.

The efficiency of electro-oxidation is strongly influenced by the value of the oxygen evolution potential (OEP). In aqueous media, a high OEP minimizes parasitic oxygen evolution reactions, thereby enhancing the selectivity and yield of hydroxyl radicals, which are the primary oxidizing species responsible for pollutant degradation. Consequently, electrode materials with high OEP values for achieving efficient and targeted glyphosate removal are examined below.

#### 3.2.1. Boron-Doped Diamond (BDD) Electrodes

BDD anodes exhibit the highest oxygen overvoltage between 2.2 and 2.6 V/SHE, and are currently one of the most effective and widely studied anodes for wastewater treatment using advanced oxidation processes [65]. Numerous works have been published showing the appropriateness of BDD anodes in anodic oxidation processes research, specifically in regard to pesticide degradation in drinking water treatment [54].

The synthesis of BDD electrodes consists of the chemical vapor deposition of a layer of boron-doped diamond on a conductive substrate (such as silicon, niobium, molybdenum or more recently, titanium) [66]. Boron doping gives the diamond layer its conductive properties by replacing certain carbon atoms in the crystal lattice. A typical boron concentration is between $10^{19}$ and $10^{21}\;{atoms\;cm}^{-3}$, which directly influences resistivity, charge carrier density as well as hydroxyl radical generation performance, essential elements in the advanced oxidation of micropollutants [54]. The advantages of the BDD anodes are their electrochemical stability, corrosion resistance in acidic environments and excellent conductive properties, which extend over a wide temperature range [67,68,69]. They can operate for several hundreds of hours, or even several years, under industrial conditions. However, their main drawback remains their high fabrication cost, mainly related to CVD deposition processes and the substrates used [70,71].

Only two papers have examined glyphosate degradation by BDD. The first study was conducted in a single-compartment electrochemical flow cell with a circular BDD anode and cathode, and tested both pure glyphosate and its commercial formulation (RoundUp^®^) [32]. The study highlighted the importance of electrolyte composition (${Na}_{2}{CO}_{3}$, ${Na}_{2}{SO}_{4}$, NaCl) and current density (10 ${mA\;cm}^{-2}$ and 100 ${mA\;cm}^{-2}$) in optimizing glyphosate degradation via conductive-diamond electrochemical oxidation. The TOC (Total Organic Carbon) concentration was evaluated during electrolysis (200 min) of synthetic wastewater polluted with 100 ${mg\;L}^{-1}$ glyphosate (0.6 ${mol\;L}^{-1}$ pure and industrial). Complete mineralization was achieved in 150 min, particularly in chloride media due to the formation of hypochlorite and other chlorine-based oxidants. Sulfate and carbonate electrolytes also contribute via persulfate and peroxocarbonate formation, though with lower efficiency (80% and 40% of TOC removal, respectively), as they are weaker oxidants than hypochlorite. However, an excess of hypochlorite may evolve into hazardous compounds like chlorate and perchlorate, which should be controlled. The study also found that higher current densities (100 ${mA\;cm}^{-2}$) increase oxidant production but are less energy-efficient due to diffusion control of glyphosate removal rate and higher ^●^OH concentration used for other reactions. No significant difference in TOC removal was obtained between pure and industrial glyphosate. Since the electrochemical degradation of glyphosate leads to the release of phosphate as a degradation product, the authors used TOC and phosphorus concentrations as controls, measured by ion chromatography, as key indicators of mineralization efficiency. Experiments showed that phosphate release reached its theoretical maximum (≈18 ${mg\;L}^{-1}$) at 10 ${mA\;cm}^{-2}$ in 15 min, regardless of glyphosate purity, with chloride-based electrolytes enabling the fastest and most efficient release, especially at low current densities. Conversely, sulfate and carbonate media showed lower efficiency, with the maximum phosphate release occurring after more than 50 min and 150 min, respectively, likely due to slower mineralization and the retention of organic nitrogen structures. Finally, analysis of nitrogen species revealed that up to 8 ${mg\;L}^{-1}$ of inorganic nitrogen (${NO}_{2}^{-}$ and ${NO}_{3}^{-}$) can be released during electrolysis. The nitrites appeared to be intermediate nitrogen species, especially in chloride and carbonate media at high current densities. In addition, electrolyte type significantly influenced ammonium formation: in chloride media, ammonium was nearly absent due to its reaction with hypochlorite forming chloramines; in carbonate media, at low current density, ammonium initially rose before declining due to ammonia stripping, as the pH during electrolysis was around 11. Conversely, at high current density, ammonia production was enhanced because the higher concentration of oxidants promoted nitrogen release. In sulfate media, particularly with industrial glyphosate, higher ammonium levels were observed due to the strong oxidizing power of peroxosulfate. Industrial glyphosate released more ammonium than pure glyphosate, suggesting additional nitrogen contributions from surfactants. This study highlights that complete mineralization of glyphosate occurs in NaCl media, where higher concentrations of oxidants are generated compared to ${Na}_{2}{CO}_{3}$ and ${Na}_{2}{SO}_{4}$.

The second study demonstrates the feasibility of a single-compartment recirculation cell at the pre-pilot scale, using a boron-doped diamond disk electrode as the anode and the electrolyte ${Na}_{2}{SO}_{4}$, for the mineralization of commercial glyphosate in acidic pH [72].

As observed similarly in the previous study, increasing the current density affects the mineralization and the biodegradability of the effluent. From 10 to 100 ${mA\;cm}^{-2}$, the electro-oxidation of glyphosate results in higher TOC removal rates, reaching up to 90% in 480 min. However, as the current density increased, the mineralization process accelerated, but the treatment of pollutants became less effective due to higher consumption of the charge used for other reactions (such as $O_{2}$ production, formation of other reactive oxidizing species, etc.) and mass transport limitations. The novelty of this study was the evaluation of the electrochemical treatment of high-concentration glyphosate solutions, specifically those resulting from the washing of pesticide storage containers. Results showed that current density had little influence on the removal efficiency of glyphosate at concentrations of 240 and 360 ${mg\;L}^{-1}$, with only a 17% increase in the time required to achieve 99% reduction. In addition, the residual glyphosate concentration was 0.7 ± 0.3 ${mg\;L}^{-1}$, still three times higher than the U.S. EPA’s recommended limit for drinking water. After 300 min of treatment, the COD removal of 83 ± 2.1% was achieved, along with improved effluent biodegradability at 80 and 100 ${mA\;cm}^{-2}$. However, below 60 ${mA\;cm}^{-2}$, biodegradability remained nearly unchanged. For the 360 ${mg\;L}^{-1}$ solution, neither treatment time nor current density significantly affected biodegradability. Although glyphosate and COD were largely removed, the authors pointed out the toxicity or the resistance to degradation of remaining organic matter. An analysis of the environmental and economic cost of the process completed this work as production of electricity is associated with high energy consumption and greenhouse gases emission. Glyphosate removal through electrochemical treatment can have varying environmental impacts depending on the energy source used. Considerations of this impact suggested that in countries relying on hydropower, the carbon footprint is relatively low, estimated at 1.3 ${kg\;CO}_{2}$ equivalent per kg of TOC removed. However, in regions where electricity is generated from non-renewable sources such as natural gas or coal, greenhouse gas emissions can increase significantly, by 170% and 439%, respectively. Conversely, using renewable energy sources like wind or solar can reduce emissions to as low as 0.3 ${kg\;CO}_{2}$ equivalent per kg TOC. Treatment costs ranged from $0.70 to $2.10 per gram of TOC removed, depending on the energy source and national subsidy policies.

#### 3.2.2. ${PbO}_{2}$ Electrodes

The OER on ${PbO}_{2}$ electrodes are between 1.8 and 2 V/SHE, which explains also the remarkable efficiency of this material in the oxidation of organic pollutants. These low-cost electrodes, compared to electrodes made from noble metals, are obtained by electrodeposition on conductive substrate, most often titanium (Ti) pre-treated thermally or chemically to improve adhesion and prevent corrosion [73]. This substrate is sometimes covered with an intermediate layer of metal oxides such as ${SnO}_{2}$ or ${TiO}_{2}$ to improve the stability of the active coating [63]. ${PbO}_{2}$ deposition is directly carried out by electrolysis of lead nitrate salt (Pb(${NO}_{3}$)_2_), at typical current densities of 10–50 ${mA\;cm}^{-2}$, in a temperature range between 60 and 80 °C for variable deposition times, which directly influence the thickness and morphology of the layer formed [73]. It has been demonstrated that the formation of the crystallized β-${PbO}_{2}$ phase, obtained as a function of the operating conditions during electrodeposition, must be favored to guarantee electrode durability and oxidation efficiency [74]. ${PbO}_{2}$ electrodes used in advanced oxidation processes exhibit lifetimes ranging from a few tens to several hundreds of hours, mainly limited by coating cracking, delamination, and substrate corrosion [75,76]. However, doping strategies can improve their stability. The study by Amadelli et al. showed that depositing ${PbO}_{2}$ on ${TiO}_{2}$ increased the electrode lifetime from 105 h to more than 280 h [77]. Nevertheless, these electrodes undergo rapid deactivation at high current densities, despite relatively low mass losses after electrolysis [78].

**Figure 3:**
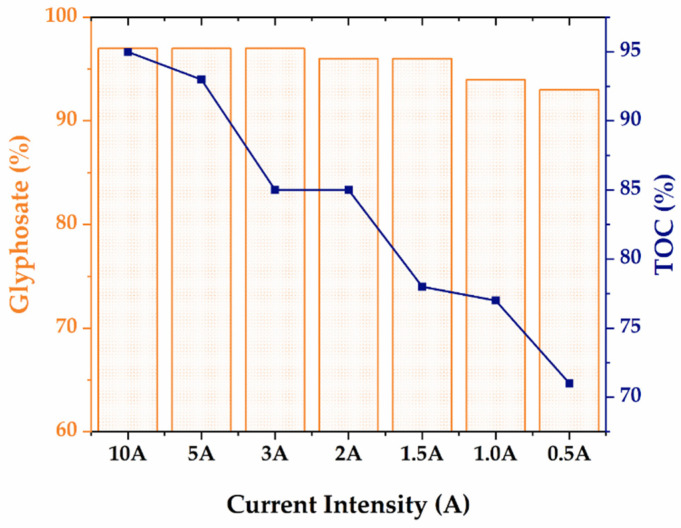
Effect of current intensity versus glyphosate degradation and mineralization rate under operating conditions: I  =  0.5–10 A (4.55–90.9 ${mA\;cm}^{-2}$, [glyphosate]_i_ =16.9 mg L^−1^, t = 180 min. (Adapted from reference [47]).

Three studies in the literature have explored the use of ${PbO}_{2}$ electrodes. The first focused on glyphosate degradation and mineralization in a sodium-based electrolyte, aiming to avoid chloride media due to the associated risk of generating toxic by-products, such as organochlorinated compounds, during electro-oxidation [47]. In this study, a Ti/${PbO}_{2}$ anode and a Ti cathode, both configured as rectangular mesh electrodes, were used. The first part of the research focused on evaluating the impact of key operational parameters, particularly the applied current intensity, which varied from 0.5 to 10.0 A, corresponding to current densities ranging from 4.55 to 90.9 ${mA\;cm}^{-2}$), treatment duration (up to 360 min), pH (from 3 to 10), and initial glyphosate concentrations (between 4.3 and 33.8 ${mg\;L}^{-1}$). Results showed that increasing current intensity led to a moderate improvement in degradation efficiency, ranging from 93.3% to 97.4%, with residual glyphosate concentrations reduced to 2.5–6.7% of the initial values. Current intensity had a more substantial impact on glycerol mineralization, as evidenced by residual TOC levels between 4.5% and 29% of the initial TOC. Additionally, the proportion of oxidizable carbon converted (TOC) increased from 71.1% to 95.5% as the current density increased from 4.55 to 90.9 ${mA\;cm}^{-2}$ (Figure 3). A minimum treatment duration of 180 min was required to achieve a 95% mineralization rate.

**Figure 4:**
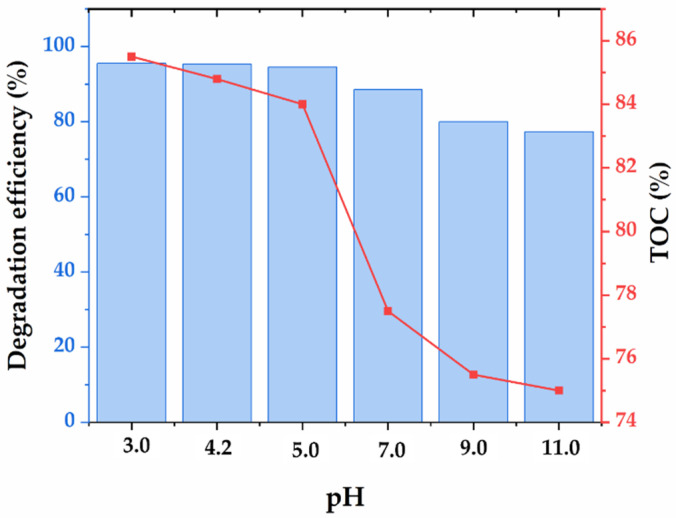
Influence of pH on glyphosate degradation and mineralization rate under operating conditions: I = 5 A, [glyphosate]_i_ = 16.9 ${mg\;L}^{-1}$, reaction time = 120 min. (Adapted from reference [47]).

The influence of pH was also examined, revealing that the highest glyphosate removal (95.5%) and TOC reduction (85.7%) occurred at pH 3 (Figure 4). A key innovation of this study lies in the use of an experimental design methodology, which enabled precise determination of the optimal operating conditions for glyphosate degradation in terms of treatment cost, with the value estimated to be 1.08 dollars per gram of glyphosate removed.

**Figure 5:**
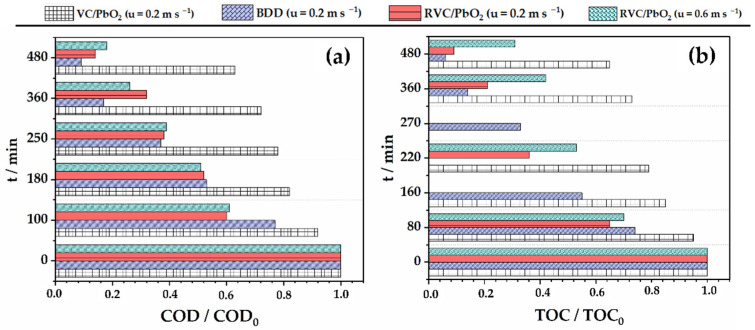
Normalized COD (**a**) and TOC (**b**) during glyphosate degradation using VC/${PbO}_{2}$ (u = 0.2 ${m\;s}^{-1}$); BDD (u = 0.2 ${m\;s}^{-1}$); RVC/${PbO}_{2}$ (u = 0.6 ${m\;s}^{-1}$); RVC/${PbO}_{2}$ (u = 0.6 ${m\;s}^{-1}$) at i = 30 ${mA\;cm}^{-2}$, T = 30 °C, in electrolyte 0.1 ${mol\;L}^{-1}$ ${Na}_{2}{SO}_{4}$. (Adapted from reference [79]).

Although ${PbO}_{2}$ exhibits favorable electrochemical and economic properties for glyphosate electro-oxidation, its potential to leach toxic ${Pb}^{2+}$ ions pose a significant environmental concern in water treatment applications. Consequently, minimizing the use of ${PbO}_{2}$ electrodes is essential. Recent advancements have focused on innovative electrode configurations to enhance performance while mitigating leaching risks. In addition, low concentrations of pesticides impose severe mass-transfer limitations that can be mitigated by three-dimensional electrodes, as the turbulence generated by a 3D matrix and its extended specific surface area enhances mass transfer. In this approach, the research group of L.A.M. Ruotolo developed a three-dimensional electrode consisting of a thin ${PbO}_{2}$ film electrodeposited onto reticulated vitreous carbon (RVC), offering improved efficiency and reduced environmental impact [79,80]. When compared to a flat ${PbO}_{2}$ electrode within an electrochemical flow reactor [79], the RVC/${PbO}_{2}$ configuration demonstrated superior oxidation kinetics, current efficiency, and energy performance, attributed to its larger surface area and improved mass transfer. As illustrated in Figure 5, glyphosate degradation based on normalized COD and TOC showed that the RVC/${PbO}_{2}$ electrode achieved a 3.9-fold and 3.0-fold increase in the pseudo-first-order rate constants for COD and TOC, respectively, compared to the flat ${PbO}_{2}$ electrode under identical hydrodynamic (0.2 ${m\;s}^{-1}$) and current density (30 ${mA\;cm}^{-2}$) conditions. Notably, the RVC/${PbO}_{2}$ electrode exhibited comparable performance to the BDD electrode, which is widely regarded as the most effective anode for glyphosate electro-oxidation. COD removal was similar for both electrodes at a flow velocity of 0.2 ${m\;s}^{-1}$ (Figure 5a), while TOC removal matched at a higher flow velocity of 0.6 ${m\;s}^{-1}$ (Figure 5b). In terms of process efficiency, both electrodes demonstrated similar current efficiency and energy consumption. Specifically, the study revealed that for the process based on RVC/${PbO}_{2}$, an energy consumption of 5.7 kWh was required to remove 95% of the TOC from 1 $m^{3}$ of wastewater containing 150 ${mg\;dm}^{-3}$ of glyphosate, as a representative concentration typically found in container wash water.

The research group further optimized the process by applying a Box–Behnken factorial design to investigate glyphosate degradation in a real effluent using the same electrochemical flow reactor configuration [80]. Increasing the flow rate enhanced mineralization kinetics, improved overall current efficiency, and reduced energy consumption by promoting greater mass transfer. Under optimized conditions, such as 30 ${mA\;cm}^{-2}$ current density, a flow rate of 3000 ${mL\;min}^{-1}$, and a temperature of 50 °C, 95% COD and 93% TOC removal were achieved after 300 min of treatment.

#### 3.2.3. Mixed Metal Oxide (MMO)

MMO electrodes constitute a diverse group of electrocatalysts, encompassing both noble metals capable of adsorbing oxygen and base metals. Their invention represented a major breakthrough in electrocatalysis, enabling the development of stable, cost-effective, and durable electrodes (lasting up to 10 years) [69], despite the inherent instability of their oxide components in acidic media caused by partial or complete reduction. The most widespread configurations, such as Ti/${TiO}_{2}$-${RuO}_{2}$, Ti/${Ta}_{2}O_{5}$-${IrO}_{2}$ and Ti/${SnO}_{2}$-${Sb}_{2}O_{5}$, are based on titanium (Ti) substrates, prized for their stability, conductivity and low cost [30]. These electrodes, also known as dimensionally stable anodes (DSA^®^, DE NORA Group, Milan, Italy) due to their remarkable properties, feature excellent corrosion resistance and a much lower degradation rate than ${PbO}_{2}$ electrodes. They enable their catalytic oxide coating to be regenerated, giving them structural longevity. The application of DSA^®^ electrodes for glyphosate degradation and mineralization remains scarcely documented in the literature [50,51,81]. MMO/DSA electrodes, particularly those based on ${IrO}_{2}$–${SnO}_{2}$–${Sb}_{2}O_{5}\;$ and ${RuO}_{2}$, exhibit good electrochemical stability and are widely used in industrial applications [82]. Their lifetime can reach several hundreds of hours, and even exceed 1000 h after coating optimization [83]. Although their electrocatalytic activity is lower than that of BDD, they provide a good balance between performance, durability, and cost. However, their degradation is mainly associated with the dissolution of active oxides and the formation of an insulating layer on the titanium substrate [84].

**Figure 6:**
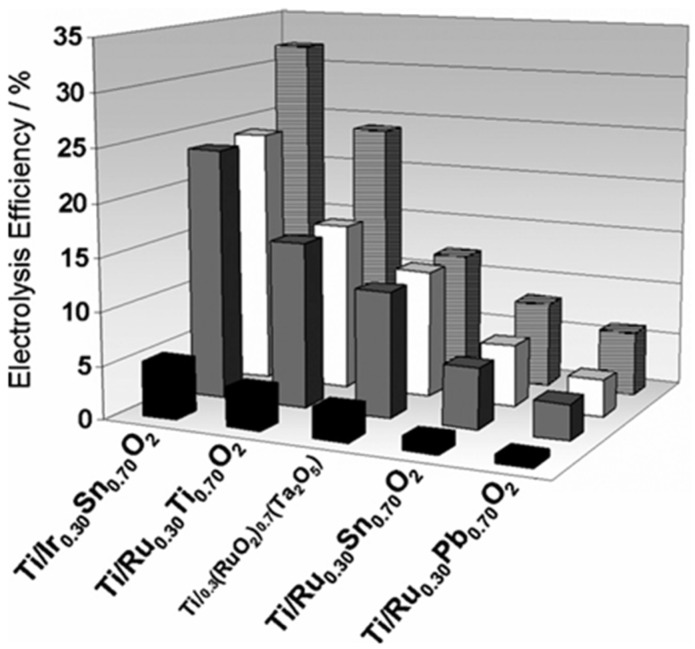
Electrolysis efficiency as a function of electrode composition. (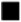) ICE, (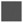) release of phosphate ions, (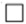) TOC removal, and (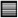) glyphosate removal from spectrophotometry data. t = 4 h, i_ap_ = 50 mA cm^−2^, μ = 1.5 (Na_2_SO_4_, pH 3). GH initial concentration: 1000 mg L^−1^. (Used with permission from Elsevier [50]).

These electrodes were applied to the electro-oxidation of glyphosate in a three-compartment electrochemical cell (50 mL) maintained at 25 ± 1 °C, in galvanostatic mode and under magnetic agitation, in solutions containing 1000 ${mg\;L}^{-1}$ of glyphosate (≈6 mM) [50]. The electrolytes used were ${Na}_{2}{SO}_{4}$ and NaCl, with current densities ranging from 30 to 100 ${mA\;cm}^{-2}$, for reaction times of 4 h and 12 h, and pH values varying from 2 to 11. Analyses performed by derivatization reaction relying respectively on a ninhydrin reaction catalyzed by ${Na}_{2}{MoO}_{4}$ at 100 °C and in a nitrosation reaction in an acidic medium. These analyses targeted residual glyphosate and its intermediates, released phosphate (${PO}_{4}^{3-}$), total organic carbon (TOC), chemical oxygen demand (COD), and current efficiency (ICE). In sulfate media, the authors observed that the anode composition strongly influenced degradation (Figure 6). The Ti/Ir_0.30_Sn_0.70_$O_{2}$ and Ti/Ru_0.30_Ti_0.70_$O_{2}$ anodes showed the best performances, with 32% and 24% of glyphosate removed after 4 h of electrolysis, respectively, whereas the other tested anodes (notably Ti/Ru_0.30_Sn_0.70_$O_{2}$, Ti/(${RuO}_{2}$)_0.70_(${Ta}_{2}O_{5}$)_0.30_, and Ti/Ru_0.30_Pb_0.70_$O_{2}$) achieved only 6–12% removal. Mineralization remained limited, with a maximum of 24% TOC removed and an ICE below 5%, indicating strong competition with the oxygen evolution reaction. The discrepancy between glyphosate disappearance and low total mineralization was attributed to the formation of recalcitrant intermediates, notably AMPA and sarcosine.

The influence of pH showed that degradation is most effective under acidic conditions (pH 2–3), while high pH markedly decreases efficiency due to the kinetic predominance of the oxygen evolution reaction. Additionally, increasing the initial glyphosate concentration (50–1000 ${mg\;L}^{-1}$) enhanced the degradation rate, likely due to greater interaction between generated oxidizing species and the target molecule. In chloride media, a marked improvement was observed in both degradation and mineralization, attributed to the formation of chlorine-based oxidizing species (${Cl}_{2}$, HClO, ${ClO}^{-}$). The degradation rate and phosphate release increased with chloride concentration, reaching an optimum at approximately 2660 ${mg\;L}^{-1}$, corresponding to nearly complete mineralization (≈91% of ${PO}_{4}^{3-}$ released). Beyond 3000 ${mg\;L}^{-1}$ of NaCl, the efficiency slightly decreased, likely due to the formation of chlorates and other secondary by-products. In this medium, the nature of the DSA^®^ anode no longer significantly affects overall performance, as all electrodes achieve nearly complete glyphosate removal and approximately 91% mineralization. According to the degradation mechanism proposed by Aquino Neto and Andrade, glyphosate undergoes two primary oxidative pathways: cleavage of the C-P bond, yielding AMPA, and cleavage of the C-N bond, producing sarcosine. These intermediates are then progressively oxidized into fully mineralized species such as ${CO}_{2}$, nitrate, and phosphate ions. Finally, regarding current density, studies showed that in sulfate media, even at 100 ${mA\;cm}^{-2}$, degradation does not exceed 50% because most of the current is consumed by the oxygen evolution reaction (OER). Conversely, in chloride media, nearly complete mineralization occurs at just 30 ${mA\;cm}^{-2}$, with no significant gains at higher intensities. The authors concluded that operating at low current density in a chloride-containing electrolyte is the most effective strategy to optimize glyphosate degradation while maintaining high current efficiency (≈80%).

**Figure 7:**
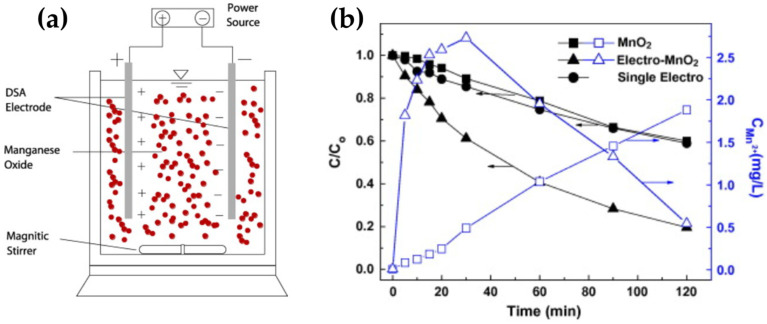
(**a**) The experimental setup for glyphosate removal by the electro-${MnO}_{2}$ process; (**b**) removal of glyphosate and release of ${Mn}^{2+}$ during the ${MnO}_{2}$, electrochemical and electro-${MnO}_{2}$ processes (C_o_ = 0.1 mM, ${MnO}_{2}$ dosage = 0.25 mM, current density = 10 ${mA\;cm}^{-2}$, pH = 5.0). (Used with permission from Separation and Purification Technology [51]).

A second study focused on the use of DSA-type ${RuO}_{2}$/${TiO}_{2}$ anodes for the oxidation of glyphosate in aqueous solution [51], whose relatively low oxygen evolution potential (1.5–1.7 V/SHE) promotes water oxidation but limits the current fraction available for the direct oxidation of organic pollutants [85]. These electrodes typically consist of a titanium substrate that is thoroughly cleaned and treated to ensure strong adhesion and optimal mechanical stability [86]. They are then coated with a catalytic layer, applied using techniques such as the sol–gel method or electroplating, which imparts high catalytic activity to the surface. This coating facilitates the formation of oxidizing species, including hydroxyl radicals, which play a crucial role in degrading organic pollutants. This study examined the electrochemical degradation of glyphosate in aqueous solution, combined with assisted oxidation using manganese dioxide (${MnO}_{2}$) as an auxiliary oxidant to improve treatment efficiency (Figure 7). ${MnO}_{2}$ promoted glyphosate oxidation and, through electrochemical regeneration, maintained a sustained oxidative capacity throughout the process. Three processes were tested: direct oxidation by ${MnO}_{2}$, electrochemical oxidation with the ${RuO}_{2}$/${TiO}_{2}$ anodes, and a combined ${MnO}_{2}$-assisted electrochemically assisted oxidation (electro-${MnO}_{2}$) process.

**Figure 8:**
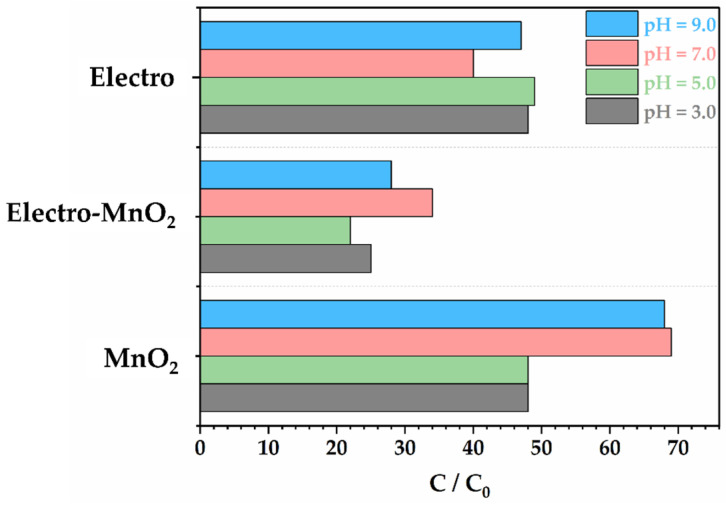
Effect of pH on glyphosate removal by the ${MnO}_{2}$ oxidation, electrochemical and electro-${MnO}_{2}$ processes. (Adapted from reference [51]).

The experiments were conducted in a 400 mL reactor containing a 0.1 M ${Na}_{2}{SO}_{4}$ solution with an initial glyphosate concentration of 0.1 mM. The authors investigated the influence of current density (0.5–10 ${mA\;cm}^{-2}$) and pH (3–9) on process performance. After 120 min, glyphosate degradation reached about 40% with ${MnO}_{2}$ or electrochemical oxidation, while it rose to 80% with the electro-${MnO}_{2}$ process. This efficiency gain was attributed to the electrochemical reoxidation of ${Mn}^{2+}$ into active ${MnO}_{2}$, which prevents its dissolution and continuously regenerates the catalyst. Regarding the influence of current density, the lowest values (0.50 and 1.00 ${mA\;cm}^{-2}$) lead to a slow degradation of glyphosate, with less than 30% and 50% removal after 120 min. At 5 ${mA\;cm}^{-2}$, the efficiency improves markedly, reaching around 60%. The best performance is observed at 10 ${mA\;cm}^{-2}$, where nearly 80% of the glyphosate is eliminated. Thus, increasing the current density enhances the production of oxidizing species and accelerates the degradation kinetics. The concentration of ${Mn}^{2+}$ increased rapidly during the first 30 min and then decreased, confirming its anodic oxidation to ${MnO}_{2}$. Regarding pH, oxidation by ${MnO}_{2}$ alone is favored in an acidic environment (pH 3–5), while in the electro-${MnO}_{2}$ process, pH exerts little influence on the degradation efficiency, reflecting the robustness of the electrochemical system (Figure 8).

**Figure 9:**
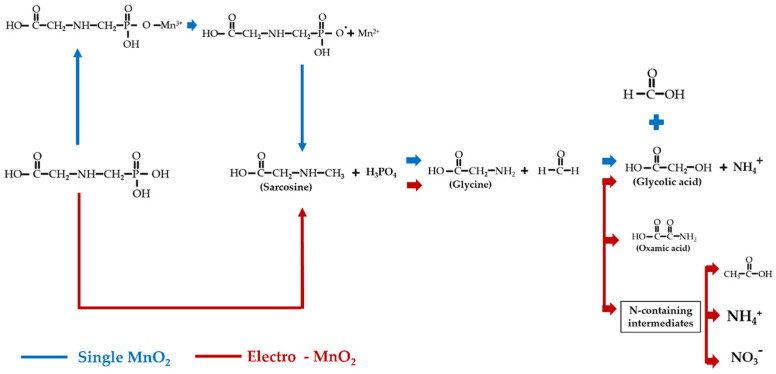
Proposed pathways for glyphosate degradation in the ${MnO}_{2}$ oxidation and electro-${MnO}_{2}$ processes. (Adapted from reference [51]).

Figure 9 compares glyphosate degradation routes in ${MnO}_{2}$ and electro-${MnO}_{2}$ processes. The main intermediates detected were sarcosine, glycine, glycolic, oxamic, acetic, and formic acids, along with ${PO}_{4}^{3-}$, ${NH}_{3}$-N, and ${NO}_{3}^{-}$-N ions. The proposed mechanism begins with C–P bond cleavage, forming sarcosine and phosphate. With ${MnO}_{2}$ alone, glycine converts to glycolic acid and ${NH}_{3}$-N, whereas in the electro-${MnO}_{2}$ system, it yields oxamic, acetic, and glycolic acids before final oxidation to ${NH}_{3}$-N and ${NO}_{3}^{-}$-N. Hydroxyl radicals generated electrochemically may drive an alternative degradation pathway. ${NH}_{3}$-N concentration in the electrochemical process increased slightly over time. In the electro-${MnO}_{2}$ process, it rose to 120 min, then declined, while in ${MnO}_{2}$ oxidation, it kept increasing. Conversely, ${NO}_{3}^{-}$-N showed an opposite trend. ${NH}_{3}$-N remained below 0.03 ${mg\;L}^{-1}$ in ${MnO}_{2}$ oxidation but reached 0.3 mg L^−1^ in electrochemical and electro-${MnO}_{2}$ processes.

This study showed that electrochemically generated hydroxyl radicals are central to this electro-assisted pathway. Overall, the electro-${MnO}_{2}$ process outperforms oxidation alone due to continuous ${MnO}_{2}$ regeneration, improved efficiency with current density, and pH insensitivity. This approach enables partial mineralization of glyphosate and its nitrogen- and phosphorus-containing intermediates, offering a promising strategy for treating recalcitrant organophosphate herbicides.

**Figure 10:**
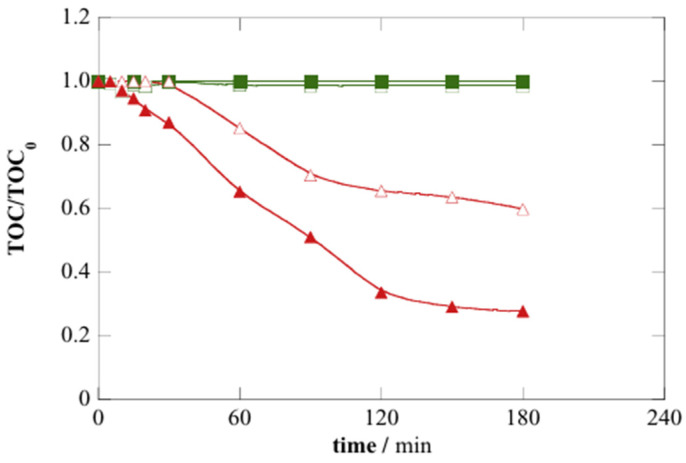
Mineralization attained during the electrochemical oxidation treatment of 100 ${mg\;L}^{-1}$. TOC solutions of (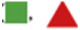) pure glyphosate and (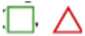) commercial pesticide formulations at j = 10 mA cm^−2^ and pH 3.0 using different supporting electrolytes: (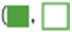) 0.15 M of Na_2_SO_4_, and (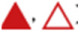) 0.15 M of NaCl. (Used with permission from Elsevier [81]).

Lima et al. also investigated DSA^®^ electrodes in glyphosate electrochemical oxidation, comparing the degradation of the pure compound with its commercial formulation containing additives [81]. Experiments were carried out in a 100 mL stirred batch reactor (800 rpm) using a Ti/${Ru}_{0.36}{Ti}_{0.64}O_{2}$ anode and a perforated stainless-steel cathode. For the tests, operating conditions included current densities of 10–40 ${mA\;cm}^{-2}$, pH 3–9, NaCl or ${Na}_{2}{SO}_{4}$ electrolytes (0.025–0.15 M), initial concentration of 100 mg of ${CL}^{-1}$ (TOC), and 3 h electrolysis. The results, presented in Figure 10, show that the nature of the electrolyte strongly influences mineralization: with ${Na}_{2}{SO}_{4}$, mineralization is limited to around 10% due to oxidation by ^●^OH radicals adsorbed on the anode surface, whereas with NaCl, mineralization exceeds 70% for pure glyphosate, owing to the formation of active chlorine species (${Cl}_{2}$, HClO, ${ClO}^{-}$) responsible for more efficient homogeneous oxidation. The study also revealed that under identical conditions (0.15 M NaCl, 40 ${mA\;cm}^{-2}$, pH 3), pure glyphosate achieved over 90% mineralization, whereas the commercial formulation reached only about 60%, due to additives consuming part of the electro-generated oxidants and reducing their availability for glyphosate degradation.

In addition, pH strongly affected glyphosate mineralization during electrochemical oxidation. TOC removal reached 77% at pH 3 versus 62% at pH 9, due to the higher oxidative capacity of ${Cl}_{2}$/HClO in acidic media compared to ${ClO}^{-}$ in alkaline conditions. Minor differences were linked to pH drift toward neutrality during treatment. Pure and commercial glyphosate showed similar behavior, with slight variations from additive speciation. Finally, increasing the current density accelerated degradation but reduced current efficiency (from 35% to 12%) and increased energy consumption (from 0.2 to 1.0 ${kWh\;g}^{-1}$ C). Beyond 30 ${mA\;cm}^{-2}$, side reactions, such as oxygen evolution or chlorate formation, limited overall efficiency. By-products generated during glyphosate oxidation were identified and quantified during the ECO treatment of pure glyphosate solutions (100 ${mg\;L}^{-1}$) in 0.15 M NaCl at pH 3.0, with an applied current density of 10 ${mA\;cm}^{-2}$ using Ti/${Ru}_{0.36}{Ti}_{0.64}O_{2}$ anodes. This analysis allowed the evolution of both organic and inorganic intermediates to be monitored and the degradation pathway to be elucidated.

Under these operating conditions, the concentration decay study showed that glyphosate degradation followed a pseudo-first-order kinetic model, with an apparent rate constant ($k_{1}$) of 3.1 $\times {\;10}^{-4}$ ($R^{2}$ = 0.992). This behavior suggests that glyphosate reacts with a quasi-constant concentration of oxidizing species continuously generated in the solution.

The process resulted in a specific electrical energy consumption of 10.25 ${kWh\;m}^{-3}$ ${order}^{-1}$, estimated using the electrical energy per order ($E_{EO}$). This value, at the lower end of the range reported for anodic materials (10–2300 ${kWh\;m}^{-3}$ ${order}^{-1}$) [87,88], suggests that the process can be up to 230 times less energy-intensive than the most demanding systems, highlighting its high energy efficiency. The $E_{EO}$ was calculated in batch mode according to Equation (5), which relates energy consumption to the electrical parameters of the cell and the apparent pseudo-first-order kinetic rate constant.

(5) $$E_{EO}\;({kWh\;m}^{-3}{order}^{-1})=\frac{6.39\times 10^{-4}•E_{cell}I}{V_{s}k_{1}}$$

where $6.39\times 10^{-4}\;$ is a conversion factor (1 h/3600 s/0.4343) and $k_{1}$ is the pseudo-first order rate constant ($S^{-1}$).

Analysis of the degradation products revealed the formation of AMPA, methylphosphonic acid, methylamine, sarcosine, as well as oxalic and formic acids as final products. The detected inorganic ions mainly included phosphate (up to 261 ${mg\;L}^{-1}$), while organic nitrogen was converted into gaseous nitrogen ($N_{2}$) through chloramination reactions, without significant accumulation of ${NH}_{4}^{+}$ or ${NO}_{3}^{-}$. These findings confirm the high efficiency of the electrochemical oxidation process for complete glyphosate degradation, producing biodegradable and non-toxic end products. However, additives in commercial formulations significantly reduce performance, underscoring the need to consider matrix effects when applying electrochemical treatments to pesticides.

#### 3.2.4. Titanium Oxide-Based Doped and Sub-Stoichiometric Electrodes

Electro-oxidation is regarded as one of the most promising advanced technologies for degrading organic micropollutants in wastewater. However, the most efficient electrodes, such as BDD or dimensionally stable anodes (DSA), are based on noble metals like ${RuO}_{2}$-${IrO}_{2}$ remain costly, limiting their large-scale application. In this context, Magnéli phases, and particularly $\;{Ti}_{4}O_{7}$, are emerging as particularly attractive alternative electrode materials. ${Ti}_{4}O_{7}$ offers excellent chemical and electrochemical stability, good electrical conductivity, and high corrosion resistance, while being significantly more economical than conventional electrodes [89,90]. The study by Ganiyu et al., conducted on the mineralization of amoxicillin, showed that after more than 200 h of operation at 60 mA, the decrease in mineralization efficiency was only about 17%, attributed to the formation of a passive layer or the partial conversion of ${Ti}_{4}O_{7}$ into a less conductive ${TiO}_{2}$ [89]. Similarly, Awesso et al. observed a limited decrease of 6.5% in mineralization after 170 h of use at 6 ${mA\;cm}^{-2}$, while glyphosate degradation remained almost unchanged. This slight loss of activity is associated with the gradual formation of a thin rutile $\;{TiO}_{2}$ layer on the anode surface, which reduces the access of hydroxyl radicals to active sites [38]. Several studies have shown that ${Ti}_{4}O_{7}\;$ anodes exhibit better stability than conventional DSA electrodes and performance close to that of BDD, while being less expensive [91,92].

The fabrication of the sub-stoichiometric titanium oxide (${Ti}_{4}O_{7}$) anode generally involves a four-step process [93]. Stoichiometric ${TiO}_{2}$, an insulator, can be converted into an n-type semiconductor by creating oxygen vacancies, achieved through thermal treatment in a reducing atmosphere or by doping with group V elements (V, Nb, Ta), which reduces Ti(IV) to Ti(III) [94]. Among the homologous oxides of the Ti-O system, the “Magnéli phases” (${Ti}_{n}O_{2n-1}$, n ≥ 3) [95] exhibit high electrical conductivity, excellent corrosion resistance, and good chemical stability. The phases ${Ti}_{3}O_{5}$, ${Ti}_{4}O_{7}$ and ${Ti}_{5}O_{9}$ are particularly interesting, with ${Ti}_{4}O_{7}$ showing a conductivity of ${166\;\Omega^{-1}\;cm}^{-1}$, far higher than that of ${TiO}_{2}$ [94], due to oxygen vacancies.

**Figure 11:**
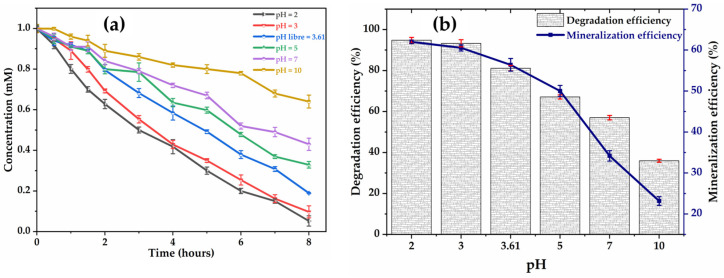
Effect of pH on: (**a**) glyphosate degradation; (**b**) mineralization. [Glyphosate] = 1 mM; [${Na}_{2}{SO}_{4}$] = 50 mM; V = 200 mL; J = 6 ${mA\;cm}^{-2}$; Θ = 20 °C. (Adapted from an open-source journal of MDPI [38]).

Commercialized as Ebonex^®^ (QuanVerge, Reno, NV, USA) sub-stoichiometric ${TiO}_{2}$, primarily ${Ti}_{4}O_{7}$, acts as a ‘non-active’ anode for water oxidation, producing hydroxyl radicals that are fewer but more reactive than those generated on BDD [96,97]. Dopant and sub-stoichiometry-modified ${TiO}_{2}$ electrodes have proven effective for degrading a wide range of organic micropollutants [89,93,98]. Only one study from 2025, by Awesso et al. [38], has investigated the electrochemical degradation of glyphosate (1 mM) on a ${Ti}_{4}O_{7}$ (Magnéli phase) anode in sulfate medium (${Na}_{2}{SO}_{4}$ 50 mM), evaluating the effects of pH (2–10) and current density (4–14 ${mA\;cm}^{-2}$) on degradation, mineralization and energy consumption. The authors demonstrated that pH plays a key role in process efficiency (Figure 11), due to its impact on hydroxyl radical generation and glyphosate adsorption on the anode surface. Acidic conditions (pH 2–3) significantly enhance glyphosate degradation, achieving 94.8% removal and 62% TOC mineralization versus 36% and 23.2% at pH 10, due to reduced oxygen evolution and stronger electrostatic attraction of protonated glyphosate to the negatively charged ${Ti}_{4}O_{7}$ surface.

**Figure 12:**
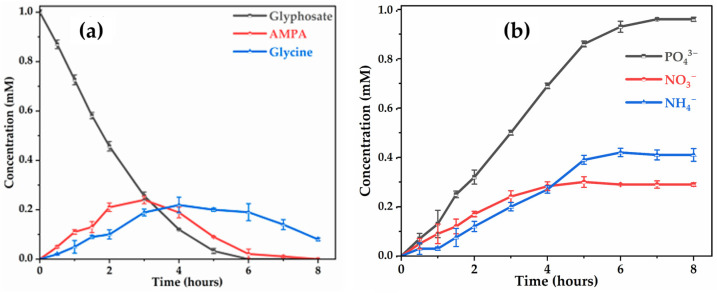
Evolution of the concentration of: (**a**) glyphosate, glycine and AMPA with time; and (**b**) phosphate, ammonium and nitrate ions. [Glyphosate] = 1 mM; [${Na}_{2}{SO}_{4}$ = 50 mM; [K_2_SO_4_] = 50 Mm (for ${NH}_{4}^{+}$); V = 200 mL; pH = 3; J = 14 mA cm^−^; Θ = 20 °C. (Adapted from an open-source journal of MDPI [38]).

The effect of current density was also investigated: glyphosate degradation increases with applied current, reaching 100% after 8 h at 14 ${mA\;cm}^{-2}$, following pseudo-first-order kinetics ($R^{2}$ = 0.90–0.98). The apparent rate rises constantly from 18.6 × $10^{-2}$ to 65.3 × $10^{-2}\;{min}^{-1}$ as the current density increases from 4 to 14 ${mA\;cm}^{-2}$, reflecting enhanced ^●^OH radical production. Mineralization follows the same trend, increasing from 43.8% to 77.8% over the same range, but the mineralization current efficiency (MCE) rapidly decreases at high current, indicating the predominance of competing reactions such as oxygen evolution. The authors therefore recommend operating at moderate current densities (4–10 ${mA\;cm}^{-2}$,) to balance performance and energy consumption. The study identified the degradation of glyphosate into AMPA and glycine intermediates (Figure 12a). AMPA degraded rapidly, disappearing within 8 h, whereas glycine persisted longer before being converted into final inorganic products ${NO}_{3}^{-}$, ${NH}_{4}^{+}$, ${PO}_{4}^{-}$ (Figure 12b), ${CO}_{2}$, and H_2_O. No sarcosine was detected, suggesting its direct conversion into glycine. After 8 h of electrolysis, 41% of the initial nitrogen was recovered as ammonium and 29% as nitrate, indicating partial mineralization. The undetected organic nitrogen (~13%) may have been lost as volatile nitrogen compounds ($N_{x}O_{y}$). Although all organic phosphorus was converted into inorganic phosphate (1 mM), a slight nitrogen deficit reflects the partial mineralization of glyphosate on the ${Ti}_{4}O_{7}$ anode.

**Figure 13:**
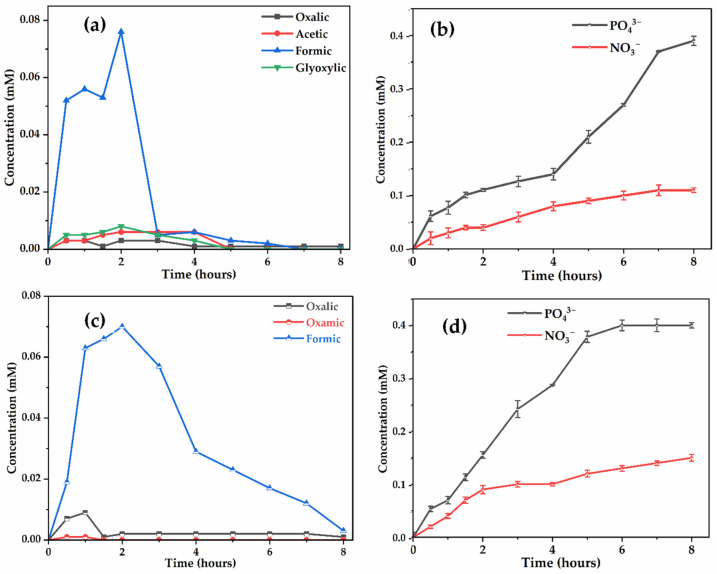
Evolution of carboxylic acids and ion concentrations on: ${Ti}_{4}O_{7}$ (**a**,**b**); BDD (**c**,**d**) during EO of retentate. [Glyphosate] = 72.3 ${mg\;L}^{-1}$ (0.41 mM); V = 200 mL; J = 10 ${mA\;cm}^{-2}$; pH = 8.45; Θ = 20 °C. (Adapted from an open-source journal of MDPI [38]).

The carboxylic acids formed (oxalic, oxamic, acetic, and formic acids) were progressively degraded, with oxamic acid being the most persistent. The overall mineralization mechanism involves the successive cleavage of C-P, C-N, and C-C bonds, leading to the nearly complete decomposition of glyphosate into harmless inorganic compounds. The study’s originality lies in coupling electrochemical treatment with membrane separation to assess the performance of a ${Ti}_{4}O_{7}$ anode in treating the retentate from nanofiltration (NF-270) of synthetic ionic water containing 0.1 mM glyphosate and various simulated salts. By combining the two processes, the treatment chain could be optimized, as NF reduces the volume of water to be treated, while electro-oxidation targets refractory compounds. This retentate, concentrated to 0.41 mM glyphosate, underwent electro-oxidation at 10 ${mA\;cm}^{-2}$ for 8 h, achieved 81.3% mineralization with 6.09 ${kWh\;g}^{-1}$ TOC energy consumption, performance comparable to BDD anode, which reached 90.5% mineralization at 5.48 ${kWh\;g}^{-1}$ TOC. During electrolysis, organic nitrogen released by covalent bond cleavage was mainly in the form of ${NH}_{4}^{+}$ and ${NO}_{3}^{-}$, with no ${NO}_{2}^{-}$ detected. These species accumulated over the 8 h treatment, whether using the ${Ti}_{4}O_{7}$ anode (Figure 13b) or BDD (Figure 13d). Specifically, 0.15 mM ${NO}_{3}^{-}$ (37% of the initial nitrogen) was found with BDD compared to 0.11 mM (27%) with ${Ti}_{4}O_{7}$. Organic phosphorus was fully converted into inorganic phosphate ions (${PO}_{4}^{-}$). Regarding the formation of carboxylic acids, Figure 13a,c, a clear difference was noted in the behavior of formic acid, a typical intermediate in glyphosate degradation [46]. On the ${Ti}_{4}O_{7}$ anode, formic acid was nearly completely removed after 7 h, while small traces (0.012 mM) remained on the BDD anode even after the same period. Overall, all carboxylic acids were effectively mineralized on both electrodes during electrolysis, with slightly faster degradation rates observed on the BDD anode compared to ${Ti}_{4}O_{7}$.

Overall, the study demonstrated that ${Ti}_{4}O_{7}$ is a high-performing, stable, and energy-efficient anode for glyphosate mineralization, particularly effective under mildly acidic conditions and at moderate current densities. Although BDD is slightly more efficient, ${Ti}_{4}O_{7}$ offers a major economic and environmental advantage due to its lower cost, chemical stability, and durability (>170 h of use). In addition, the study showed that coupling nanofiltration with electro-oxidation reduces both toxicity and contaminated water volume, lowering bacterial inhibition from 9.8% to under 5% after treatment. The authors suggest that future integration with electro-Fenton processes could further enhance mineralization and energy efficiency, paving the way for sustainable and economically viable management of pesticide-contaminated waters.

To finalize the section devoted to the efficiency of glyphosate electro-degradation according to the nature of the electrode, Table 3 briefly summarizes the major advantages and disadvantages of the different electrodes examined.

## 4. Conclusions and Perspectives

Electrochemical anodic oxidation has demonstrated significant potential as an advanced treatment technology for glyphosate-contaminated water, achieving near-complete mineralization under optimized conditions. The efficiency of this process is strongly governed by the nature of the electrode material and operational parameters. Boron-doped diamond (BDD) electrodes remain the benchmark due to their high oxygen evolution overpotential and ability to generate abundant hydroxyl radicals, enabling rapid and complete mineralization. However, their high cost limits large-scale applications. Alternatives such as the following electrodes offer competitive performance at substantially lower cost: $PbO_{2}$ is effective and economical for the electrooxidation of glyphosate, but its environmental impact and sustainability are major obstacles. DSA electrodes are robust, cost-effective, and widely used, but their lower oxidation power and risk of by-product formation make them less ideal for complete glyphosate mineralization compared to advanced electrodes like BDD and ${Ti}_{4}O_{7}$. Electrochemical oxidation using Magnéli phase ${Ti}_{4}O_{7}$ emerges as a highly promising and competitive strategy for glyphosate degradation and substantial mineralization, owing to its ability to generate reactive oxygen species at the electrode surface. Beyond its intrinsic reactivity, this material combines strong electrochemical performance, robustness, and operational reliability, making it particularly well-suited for advanced oxidation processes. While benchmark anodes such as boron-doped diamond remain a reference in terms of absolute performance, ${Ti}_{4}O_{7}$ offers a far more balanced profile by combining high oxidation efficiency with significantly improved economic and practical feasibility. Its fabrication from abundant and accessible precursors, together with less demanding production routes, provides a decisive advantage for large-scale implementation and real-world applications. Overall, ${Ti}_{4}O_{7}$ stands out as a strategic electrode material that successfully bridges the gap between performance, stability, and cost.

Operational factors, including acidic pH (2–3), moderate current densities (10–30 ${mA\;cm}^{-2}$), and chloride-containing electrolytes (with chlorine-based oxidants form both near the electrode and in the bulk solution, reducing mass transport limitations), further enhance degradation efficiency by promoting hydroxyl radical formation and secondary oxidant generation. Despite these advantages, challenges persist, notably mass transfer limitations, parasitic oxygen evolution reactions at high current densities, and the formation of chlorinated by-products in chloride media. Additionally, energy consumption and carbon footprint vary significantly depending on electrode type and electricity source, underscoring the need for sustainable integration strategies. Importantly, current density cannot be considered an independent operational parameter, as it is intrinsically coupled with mass transport, charge carrier mobility, and particularly the overpotential governing electrode kinetics; therefore, future studies should integrate electrochemical kinetic modeling (e.g., Tafel and Butler–Volmer analyses) with degradation profiles to better differentiate surface- and transport-controlled regimes. In addition, although hydroxyl radicals are widely recognized as the main oxidative species, a quantitative description of reactive oxygen species evolution as a function of potential and current density remains lacking, and advances in operando diagnostic techniques (e.g., EPR, SECM, spectroelectrochemistry) will be essential to elucidate these mechanisms, especially for complex electrode materials.

Future research should prioritize the development of cost-effective and durable electrode materials, particularly Magnéli-phase ${Ti}_{4}O_{7}$ and hybrid configurations incorporating catalytic coatings or nanostructures to enhance radical generation and mass transfer. Although only a limited number of studies have investigated 3D electrode configurations within the emerging field of Reactive Electrochemical Membranes (REMs), addressing mass-transfer limitations clearly represents a major opportunity to enhance glyphosate degradation performance. The development of flow-through electrochemical reactors, operating with REMs, should therefore be strongly encouraged, as these systems force the liquid phase to permeate the porous electrode structure, ensuring direct convective delivery of pollutants to electroactive sites. By promoting higher mass-transfer rates and minimizing diffusion constraints, flow-through architectures offer markedly superior efficiency compared with conventional planar, flow-by electrochemical cells.

Comprehensive studies on by-product formation, especially chlorinated and nitrogenous species, are essential to ensure safe effluent discharge and compliance with environmental standards. Energy optimization strategies, including modeling of current efficiency under real operating conditions and coupling with renewable energy sources, will be vital to minimize operational costs and greenhouse gas emissions. Real-world applications should be systematically considered to validate performance on industrial effluents and high-strength glyphosate waste streams, paving the way for the practical implementation of electrochemical anodic oxidation as a sustainable solution for glyphosate-contaminated waters.

Finally, the comparative assessment of competing treatment technologies reveals that no single process offers an optimal balance of cost, scalability, and mineralization efficiency for glyphosate-contaminated wastewater. Adsorption remains economically attractive due to its simplicity and low operating cost, yet its lack of selectivity, rapid sorbent saturation, and the need for post-treatment disposal limit its viability as a standalone solution at an industrial scale. Biological processes offer the greatest economic advantage, with low energy demand and suitability for large volume treatment, but their performance is hindered by slow kinetics, sensitivity to wastewater composition, and incomplete mineralization (especially poor degradation of AMPA), which restricts their applicability to real industrial effluents without prior conditioning or subsequent polishing steps. In contrast, anodic electro-oxidation exhibits strong destructive capability and can achieve near-complete mineralization under optimized conditions, yet its industrial deployment is constrained by high electrical energy consumption, electrode fouling, and mass transfer limitations. Overall, these findings highlight that integrated treatment pathways, combining low-cost bulk removal (biological or adsorption) with highly efficient oxidative refinement (anodic electrochemical process), could represent the most economically and operationally viable strategies for the scalable and sustainable removal of glyphosate and its recalcitrant metabolites.

## Figures and Tables

**Table 1:** Performance comparison of conventional and electrochemical treatment methods for water pollutant removal.

Treatment Method	Advantages	Challenges
Physicochemical	Fast treatment kinetics High phosphorus removal efficiency Simple operation Effective for concentrated effluents Possibility of adsorbent reuse	Sludge generation Chemical consumption Adsorbent saturation Secondary waste management Limited removal of dissolved organic phosphorus
Biological	Eco-friendly process Low operational cost Nutrient recovery potential Low chemical requirement Suitable for large-scale systems	Large footprint Slow kinetics Sensitive to environmental conditions Risk of eutrophication Limited efficiency for recalcitrant pollutants
Electrochemical	High oxidation efficiency Effective for refractory pollutants Low chemical input Compact reactor design Easy automation Possible complete mineralization	High energy demand Electrode fouling Expensive electrode materials Mass transfer limitations Possible toxic by-product formation

**Table 2:** Comparison of the advanced oxidation processes for glyphosate removal.

Treatment Technique	Methods to Produce Radicals	Advantages	Limitations
Electrochemical oxidation	Anodic electrical energy	Outstanding performance with high amounts of wastewater Very clean method User-friendly	Mass transfer limitation Limited electrode lifespan
Photolysis-assisted oxidation	UV light	Outstanding performance with high amounts of wastewater	Complicated due to low UV penetration in important media Difficult to scale up
Ozonation oxidation	Ozone	Outstanding performance at low concentration levels	Limitation of ozone mass transfer Major maintenance Instability of ozone Solubility challenges prompt a complex mixing method
Fenton-assisted oxidation	Decomposition of $H_{2}O_{2}$ into •OH radicals in the presence of ferrous ions	Outstanding performance at low concentration levels User-friendly Unlimited mass transfer	Acidic pH requirement Recycling of ferrous ion Generation of sludge requiring additional treatment
Photoelectrochemical oxidation	UV light and Anodic electrical energy	Higher degradation and mineralization efficiency Complete mineralization Faster kinetics and enhanced reaction pathways	Synergy depends on conditions: Antagonistic or weak effects at low current densities or with certain electrolytes (e.g., carbonate, chloride) Requires high energy consumption Complex process control: optimization of current and light
Integrated adsorption–anodic electro-oxidation process	Anodic electrical energy	Efficient pollutant pre-concentration step In situ degradation Compatibility with high-performance anodes Combined destruction and reuse process that promotes the circular economy	Strong dependence on operating conditions (pH and electrolytes) for the adsorption step Mass transfer limitations: diffusion of pollutants between carbon pores and electroactive zones can limit reaction rates

**Table 3:** Advantages and disadvantages of some common anode materials in AOPs.

Electrode Type	Advantages	Disadvantages
Mixed metal oxide electrodes, also called Dimensionally Stable Anodes (DSA)	Good conductivity properties, Acceptable price, Possibility to regenerate catalytic oxide coating Robust and dimensionally stable Cost-effective and scalable Commonly used in large-scale electrochemical processes, making them suitable for wastewater treatment.	Sensitivity to corrosion in acidic medium (for ${RuO}_{2}$ and ${IrO}_{2}$), and coating degradation in complex environments. Quite expensive due to the chemical composition Risk of chlorinated by-products in chlorine media, which can lead to secondary pollution. Less efficient for complete mineralization
BDD	Highest overpotential towards OER (2.2 and 2.6 V/SHE) Excellent conducting properties even at low temperatures High electrochemical stability and corrosion resistance in acidic environments High production of ^●^OH radicals Complete mineralization of glyphosate in chlorite medium at low current density (10 ${mAcm}^{-2}$)	Expensive due to the complex fabrication process and chemical composition Reduced efficiency in diluted solutions
${PbO}_{2}$	Relatively low cost High overpotential towards OER (1.8–2 V/SHE) Good electrochemical stability Relatively high ability to mineralize organics Easy to deposit on conductive substrate (Ti, graphite) by electrodeposition, which facilitates manufacturing.	Toxicity (Pb leaching) Environmental concerns Under harsh conditions (strong currents, aggressive environments), limited stability Limited applicability for industrial wastewater treatment
${Ti}_{4}O_{7}$	Relatively low cost due to chemical composition Good electrical conductivity and high corrosion resistance Easy to manufacture and to shape in the form of a porous reactive electrochemical membrane (REM)	Limited chemical and electrochemical stability when using high current density Fouling and passivation: In real water matrices, deposits and organic matter can reduce the catalytic activity of ${Ti}_{4}O_{7}$.

## Data Availability

No new data were created or analyzed in this study. Data sharing is not applicable to this article.
